# Nanoparticle-based delivery of siDCAMKL-1 increases *microRNA-144 *and inhibits colorectal cancer tumor growth via a Notch-1 dependent mechanism

**DOI:** 10.1186/1477-3155-9-40

**Published:** 2011-09-19

**Authors:** Sripathi M Sureban, Randal May, Fadee G Mondalek, Dongfeng Qu, Sivapriya Ponnurangam, Panayotis Pantazis, Shrikant Anant, Rama P Ramanujam, Courtney W Houchen

**Affiliations:** 1 Department of Medicine, University of Oklahoma Health Sciences Center, Oklahoma City, Oklahoma 73104, USA; 2 Department of Veterans Affairs Medical Center, Oklahoma City, Oklahoma 73104, USA; 3 OU Cancer Institute, Oklahoma City, Oklahoma 73104, USA; 4 Swaasth, Inc., 800 Research Parkway Suite 350, Oklahoma City, OK 73104, USA; 5 ADNA Inc., Dublin, Ohio 43017, USA; 6 Kansas University Medical Center, Kansas City, KS 66160, USA

**Keywords:** DCAMKL-1, *miR-144*, microRNA, siRNA, notch signaling, nanoparticles, HCT116, tumor xenograft, cancer stem cells

## Abstract

**Background:**

The development of effective drug delivery systems capable of transporting small interfering RNA (siRNA) has been elusive. We have previously reported that colorectal cancer tumor xenograft growth was arrested following treatment with liposomal preparation of siDCAMKL-1. In this report, we have utilized Nanoparticle (NP) technology to deliver DCAMKL-1 specific siRNA to knockdown potential key cancer regulators. In this study, mRNA/miRNA were analyzed using real-time RT-PCR and protein by western blot/immunohistochemistry. siDCAMKL-1 was encapsulated in Poly(lactide-*co*-glycolide)-based NPs (NP-siDCAMKL-1); Tumor xenografts were generated in nude mice, treated with NP-siDCAMKL-1 and DAPT (γ-secretase inhibitor) alone and in combination. To measure *let-7a *and *miR-144 *expression *in vitro*, HCT116 cells were transfected with plasmids encoding the firefly luciferase gene with *let-7a *and *miR-144 *miRNA binding sites in the 3'UTR.

**Results:**

Administration of NP-siDCAMKL-1 into HCT116 xenografts resulted in tumor growth arrest, downregulation of proto-oncogene c-Myc and Notch-1 via *let-7a *and *miR-144 *miRNA-dependent mechanisms, respectively. A corresponding reduction in *let-7a *and *miR-144 *specific luciferase activity was observed *in vitro*. Moreover, an upregulation of EMT inhibitor *miR-200a *and downregulation of the EMT-associated transcription factors ZEB1, ZEB2, Snail and Slug were observed *in vivo*. Lastly, DAPT-mediated inhibition of Notch-1 resulted in HCT116 tumor growth arrest and down regulation of Notch-1 via a *miR-144 *dependent mechanism.

**Conclusions:**

These findings demonstrate that nanoparticle-based delivery of siRNAs directed at critical targets such as DCAMKL-1 may provide a novel approach to treat cancer through the regulation of endogenous miRNAs.

## Background

Colorectal cancer is the second most common tumor type in the US and is the third leading cause of cancer-related mortality, accounting for nearly 9% of all cancer-related deaths [1]. In the gut, tumorigenesis arises from the stem cell population located near the base of intestinal and colonic crypts [2]. We have recently reported that the putative intestinal stem cell marker musashi-1 (Msi-1) regulates Notch-1 in colorectal cancer [3]. Msi-1 is a positive regulator of Notch signaling through translational repression of m-numb mRNA (an inhibitor of Notch signaling) [4].

The novel putative intestinal and pancreatic stem cell marker DCAMKL-1 [5-7], a microtubule-associated kinase is upregulated in colorectal and pancreatic cancers and plays a functional role in tumorigenesis through regulation of the tumor suppressor microRNAs (*let-7a*, *miR-200a *and *miR-144*) and their downstream targets such as c-Myc, KRAS, ZEB1, ZEB2 and Notch-1 [8,9].

Post-transcriptional silencing of disease-associated genes using exogenous short interfering RNA (siRNA) is an exciting new strategy to treat various human diseases [10,11]. However, the clinical application of siRNA has been hindered by its rapid degradation, nonspecific distribution and poor cellular uptake [12]. Consequently, delivery systems capable of protecting and transporting siRNA through both extracellular and intracellular barriers to reach the site of action in the cytosol are required for successful development of siRNA-based therapeutics [13]. Nonviral siRNA delivery systems such as cationic lipids, cationic polymers, and cell-penetrating peptides have been studied intensively. However, the use of cationic vectors for clinical applications has been severely limited by their high toxicity, low serum stability, nonspecific immune-stimulating effects, and poor biodegradability [14].

The use of poly(lactide-*co*-glycolide) or PLGA nanoparticles (NPs) has emerged as a powerful potential methodology for carrying both small and large molecules of therapeutic importance, as well as scaffolds for tissue engineering applications. This utility derives primarily from: (a) physiologic compatibility of PLGA and its monomers, polyglycolic acid (PGA) and polylactic acid (PLA), all of which have been established to be safe for human use for more than 30 years in various biomedical applications including drug delivery systems [15]; (b) the commercial availability of a variety of PLGA formulations which allow for control over the rate and duration of molecules released for optimal physiological response [16,17]; (c) the biodegradability of PLGA materials, which provides for sustained release of the encapsulated molecules under physiological conditions, and conversion of PLGA to nontoxic, low-molecular-weight products that are readily eliminated [18]; and (d) the ability to manufacture PLGA nanoscale particles (< 500 nm) for potential evasion of the immune phagocytic system or fabrication into microparticles on the cellular scale for targeted delivery of drugs or as antigen-presenting systems [19]. This unique combination of properties coupled with flexibility during fabrication has led to interest in modifying the PLGA surface for specific attachment to cells or organs in the body [20,21] to support drug delivery and tissue engineering applications.

We have recently demonstrated that targeted inhibition of DCAMKL-1 resulted in induction of key tumor suppressor miRNAs and subsequent abrogation of several critical oncogenic pathways [8,9]. In this report, we use PLGA NPs as a delivery vehicle for siDCAMKL-1 to study the effect on colon cancer cells both *in vitro *and *in vivo*.

## Results

### DCAMKL-is increased in various human cancers

**Figure 1:**
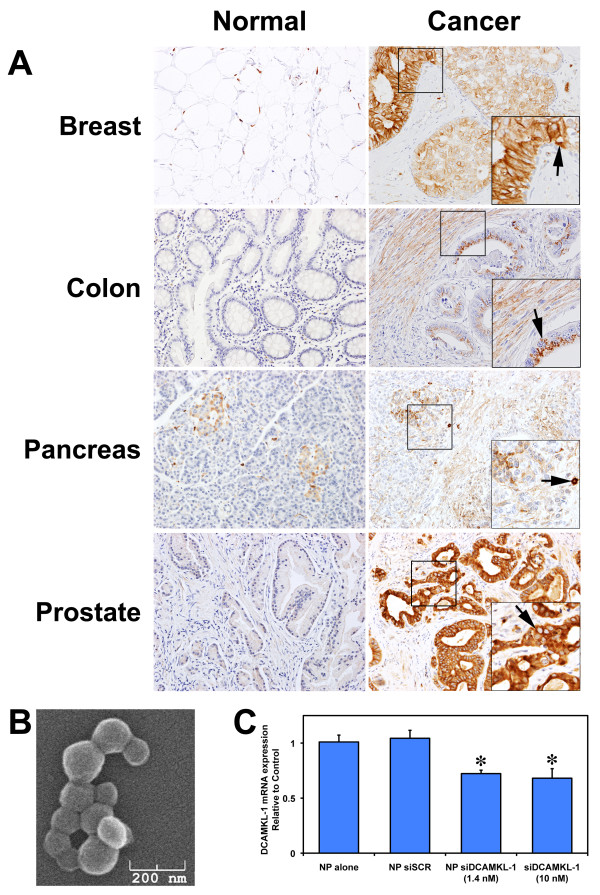
**DCAMKL-1 is overexpressed in various human tumors**. (*A*) Immunohistochemistry of DCAMKL-1 (brown) in different human normal and tumor tissues. Arrows indicate the cells positive for DCAMKL-1, insets are magnified images. (*B*) Electron microscopic photograph of siDCAMKL-1 encapsulated PLGA-Nanoparticle. (*C*) DCAMKL-1 specific siRNA (siDCAMKL-1) encapsulated in NPs (NP siDCAMKL-1), but not scrambled siRNA (siSCR) encapsulated in NPs (NP siSCR) decreases DCAMKL-1 mRNA expression in HCT116 cells. siDCAMKL-1 was also transfected into HCT116 cells using Ambion transfection reagent. For *C*, values in the bar graph are given as average ± SEM, and *asterisks *denote statistically significant differences (*P *< 0.01) compared with control (NP alone).

We performed immunohistochemical analyses on human multi-cancer tissue microarrays (Tissue Array Network, Rockville, MD). Immunoreactive minimal or no DCAMKL-1 was detected in normal breast, colon, pancreas and prostate (Figure 1A - left panels), whereas an increased expression of DCAMKL-1 was detected in the cytoplasm of epithelial cells and in the stroma of breast, colon, pancreatic and prostate cancers (Figure 1A - right panels; brown staining, indicated by arrows).

### Nanoparticle-based siRNA delivery

PLGA NPs have primarily been used as a vehicle for pharmaceutical delivery of nucleotides, hormones, or drugs to target tissues [22]. Here, we employed a PLGA NP-based siRNA delivery approach as an alternative to liposomal encapsulation, which we previously used [3,9,23]. Figure 1B demonstrates a scanning electron micrograph of PLGA nanoparticle-encapsulated siRNA. Total RNA isolated from HCT116 cells treated with NPs (containing 1.4 nM of siDCAMKL-1 and 10 nM of siSCR) separately and transfection reagent (Ambion) for 48 h was subjected to real-time RT-PCR analysis for DCAMKL-1. We observed a reduction in DCAMKL-1 mRNA following treatment with siDCAMKL-1 alone or encapsulated in NPs; these results are similar to our previous experimental observations [9]. Less siRNA (7-fold) was required, however, when delivered encapsulated in PLGA-NPs (Figure 1C).

### DCAMKL-1 knockdown and DAPT-induced Notch inhibition blocks tumor progression

**Figure 2:**
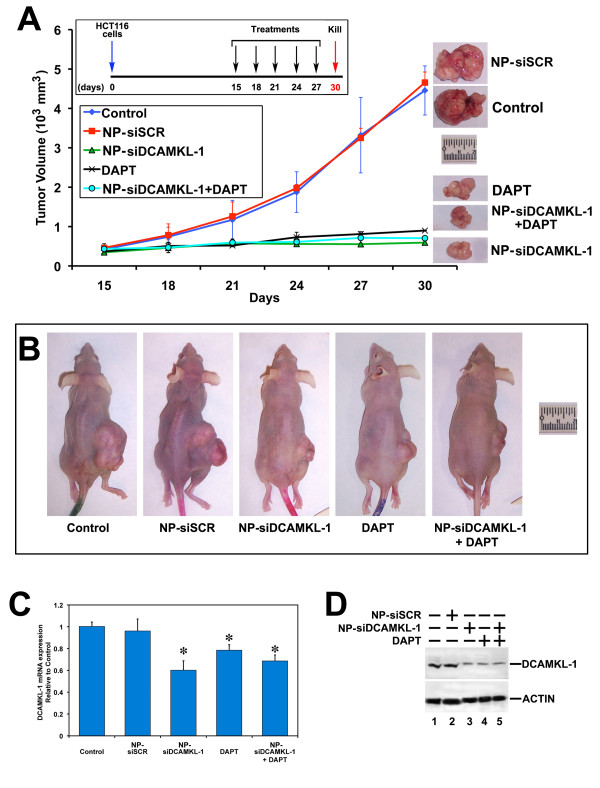
**Knockdown of DCAMKL-1 and Notch inhibition arrests tumor growth**. (*A*) HCT116 cells were injected into the flanks of athymic nude mice and tumors were allowed to develop for 15 days. NP encapsulated siRNAs (siDCAMKL-1 and siSCR) were injected directly into the tumors. Different goups of animals were injected with DAPT in corn oil (i.p) and NP-siDCAMKL-1+DAPT. Treatments were started on day 15 and given every third day for a total of five injections (*inset*). Tumors were excised at day 30 and the tumor volumes are represented above from the data collected at the time of every injection. A representative excised tumor at day 30 is shown on the right. (*B*) Representative photograph of mice bearing the tumors from each group are shown. (*C*) The expression of DCAMKL-1 mRNA in the tumors quantitated by real-time RT-PCR. (*D*) DCAMKL-1 protein expression was assessed on tumor samples by Western blot analysis. For *C*, values are given as average ± SEM, and *asterisks *denote statistically significant differences (*P *< 0.01) compared with control (NP alone).

We generated tumor xenografts by injecting HCT116 cells (6 × 10^6^) subcutaneously into the flanks of NCr-nu/nu athymic nude mice (see Methods). Starting on day 15, post-injection of cells, the resulting tumors were treated with NPs alone (control), NP-siSCR, NP-siDCAMKL-1, DAPT alone, or NP-siDCAMKL-1+DAPT on every third day for a total of five doses (Figure 2A). Tumor volumes were measured at the time of treatments using a caliper and tumor weight was measured after death^3, 8, 24^. Administration of NP-siDCAMKL-1, DAPT, and NP-siDCAMKL-1+DAPT resulted in a significant reduction (*p *< 0.01) in tumor volume and weight compared with either the control (NPs-alone) or NP-siSCR-treated tumors (Figure 2A, B and Additional File 1, Figure S1). mRNA analysis demonstrated a significant downregulation (*p *< 0.01) of DCAMKL-1 mRNA compared to control or NP-siSCR treated tumors (Figure 2C). Similarly, a reduction in DCAMKL-1 protein was observed in tumors treated with NP-siDCAMKL-1, DAPT, and NP-siDCAMKL-1+DAPT compared to control or NP-siSCR treated tumors (Figure 2D). These data taken together suggest that inhibition of DCAMKL-1 and/or Notch-1 results in HCT116 tumor xenograft growth arrest.

### DCAMKL-1 mediated regulation of *let-7a *miRNA

**Figure 3:**
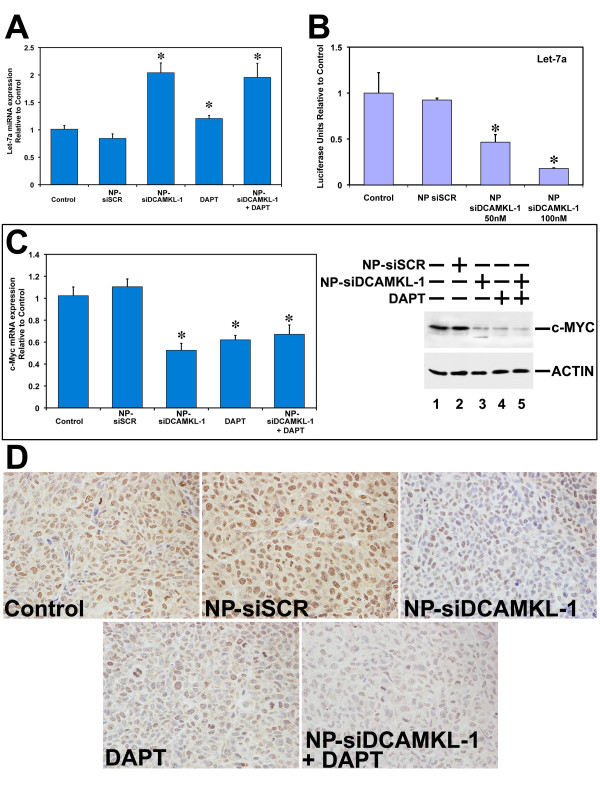
**Inhibition of DCAMKL-1 and Notch downregulates c-Myc via inducion of *pri-let-7a *miRNA**. (*A*) Quantitative real-time RT-PCR analysis for *pri-let-7a *miRNA in tumor xenografts. siRNA-mediated knockdown of DCAMKL-1 and DAPT-mediated inhibition of Notch signaling results in increased expression of *pri-let-7a *miRNA. (*B*) NP-siDCAMKL-1-mediated knockdown of DCAMKL-1 decreases luciferase activity (luciferase units) following transfection with plasmid encoding luciferase containing *let-7a *binding site in HCT116 cells. (*C*) A decreased expression of c-Myc mRNA and protein was observed in HCT116 tumor xenografts following knockdown of DCAMKL-1 and Notch. (*D*) Decreased c-Myc expression (brown) in NP-siDCAMKL-1, DAPT and NP-siDCAMKL-1+DAPT treated tumors compared with controls by immunohistochemical analysis. For bar graph in *A*, *B *and *C*, values are given as average ± SEM, and *asterisks *denote statistically significant differences (*P *< 0.01) compared with control (NP alone).

To determine whether NP-based delivery of siDCAMKL-1 regulated *let-7a *miRNA as described previously [8,9], HCT116 tumor xenografts (treated with NPs alone, NP-siSCR, NP-siDCAMKL-1, DAPT alone, and NP-siDCAMKL-1+DAPT) were analyzed for *pri-let-7a *miRNA expression by real-time RT-PCR and normalized using *pri-U6 *miRNA. Compared to control and NP-siSCR treated tumors, there was a ~2-fold increase in *pri-let-7a *miRNA expression in DCAMKL-1 siRNA-treated tumors (Figure 3A). This was similar to our previously published data using DOPC-mediated delivery of siDCAMKL-1 into HCT116 tumor xenografts. Furthermore, we observed a statistically significant increase in the expression of *pri-let-7a *miRNA following treatment with DAPT or NP-siDCAMKL-1+DAPT compared to NPs alone or NP-siSCR treated tumors (Figure 3A). We next performed a luciferase reporter gene assay to quantitatively measure the effect of siRNA-mediated downregulation of DCAMKL-1 on *let-7a *miRNA. HCT116 cells were transfected with a plasmid containing firefly luciferase gene with a complementary *let-7a *binding site at the 3' UTR. Following transfection, cells were treated with NPs alone, NP-siSCR, or NP-siDCAMKL-1 and were subjected to luciferase activity measurement. A dose-dependent reduction in luciferase activity was observed in cells treated with 50 or 100 nM of NP-siDCAMKL-1 compared to control or NP-siSCR (Figure 3B). These data suggest that knockdown of DCAMKL-1 using NP-encapsulated siDCAMKL-1 results in downregulation of *let-7a *miRNA downstream targets in HCT116 cells. Subsequently, we evaluated the expression of proto-oncogene c-Myc (a downstream target to *let-7a *miRNA) [9] in the HCT116 tumor xenografts. In tumors treated with NP-siDCAMKL-1, DAPT, or NP-siDCAMKL-1+DAPT compared to NPs alone (control) or NP-siSCR, we observed a ~50% reduction in c-Myc mRNA estimated using real-time RT-PCR and a reduction in c-Myc protein revealed by Western blot analysis (Figure 3C). This downregulation was associated with reduced c-Myc protein as determined by immunohistochemical analyses (Figure 3D).

### DCAMKL-1 regulates Notch-1 via a *miR-144 *dependent mechanism

**Figure 4:**
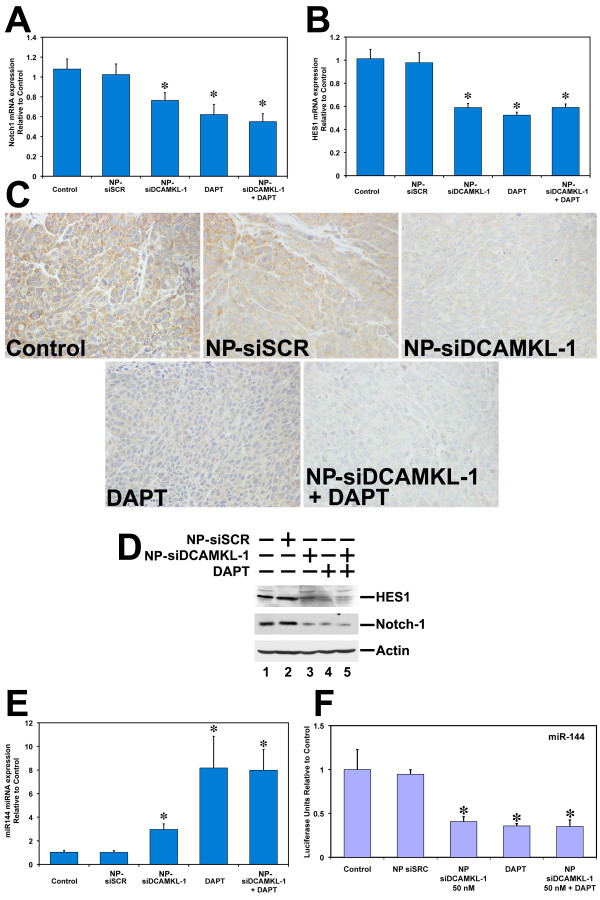
**Knockdown of DCAMKL-1 and treatment with DAPT downregulates Notch-1 via *miR-144***. siRNA-mediated knockdown of DCAMKL-1 and DAPT treatment decreases Notch-1 (*A*) and HES1 mRNA (*B*) in HCT116 tumor xenografts. Decreased Notch-1 protein expression (brown) in NP-siDCAMKL-1, DAPT and NP-siDCAMKL-1+DAPT treated tumors compared with controls by immunohistochemical analysis (*C*) and by Western blot analysis (*D*). Knockdown of DCAMKL-1 and treatment with DAPT results in increased expression of *pri-miR-144 *miRNA in tumor xenografts (*E*) and decreases luciferase activity (luciferase units) following transfection with plasmid-encoding luciferase containing the *miR-144 *binding site in HCT116 cells. For bar graph in *A, B, E *and *F*, values are given as average ± SEM, and *asterisks *denote statistically significant differences (*P *< 0.01) compared with control (NP alone).

Upregulation of Notch receptors and their ligands have been described in several cancers including cervical, lung, colon, head and neck, renal and pancreatic cancer [24-28]. Given the potential roles of Notch signaling in adult stem cell regulation and tumorigenesis [29], we determined the effect of NP-siRNA-mediated knockdown of DCAMKL-1 on Notch-1 expression in HCT116 cell tumor xenografts. We observed a significant reduction in Notch-1 mRNA in tumor xenografts treated with NP-siDCAMKL-1, DAPT, and NP-siDCAMKL-1+DAPT compared to NPs alone (control) or NP-siSCR treated tumors (Figure 4A). A reduction in the expression of Notch-1 protein was observed by immunohistochemical (Figure 4C) and Western blot analyses (Figure 4D).

Hairy and Enhancer of Split 1 (HES1) is a transcription factor and target gene of the canonical Notch signaling pathway [30]. It has been confirmed that HES1 is the downstream effector of Notch-1 and Hedgehog signaling pathways and that these pathways are frequently upregulated in tumors [31]. Compounds that inhibit these pathways induce differentiation and apoptosis in cancer cells; several are currently in clinical trials [32]. We observed a significant reduction in HES1 mRNA (Figure 4B) and protein (Figure 4D) in the tumor xenografts treated with NP-siDCAMKL-1, DAPT, and NP-siDCAMKL-1+DAPT compared to control or NP-siSCR treated tumors.

We previously found a predicted binding site for *miR-144 *in the Notch-1 3' UTR (at the 189^th ^base pair) (http://www.microrna.org: a resource for microRNA targets and expression) (Additional File 2, Figure S2) [8]. To investigate the role of DCAMKL-1 in regulating Notch-1 via *miR-144 *miRNA in colorectal cancer, HCT116 tumor xenografts were analyzed for *pri-miR-144 *miRNA expression by real-time RT-PCR. Compared to control and NP-siSCR-treated tumors, there was a 3-fold increase in *pri-miR-144 *miRNA expression in NP-siDCAMKL-1-treated tumors (Figure 4E). These data suggest that DCAMKL-1 negatively regulates *pri-miR-144 *miRNA in human colorectal cancer cells. Furthermore, tumors treated with DAPT and NP-siDCAMKL-1+DAPT demonstrated an 8-fold increase in *pri-miR-144 *miRNA expression compared to control and NP-siSCR-treated tumors.

To evaluate these findings quantitatively, we performed a luciferase reporter gene assay using HCT116 cells containing the firefly luciferase gene with a complementary *miR-144 *binding site in the 3'UTR. A statistically significant reduction in luciferase activity was observed following DCAMKL-1 knockdown (Figure 4F), indicating that DCAMKL-1 may be a posttranscriptional regulator of *miR-144 *miRNA downstream targets in colorectal cancer. Taken together, these data strongly suggest that Notch-1 is a downstream target of *miR-144 *miRNA and that DCAMKL-1 regulates posttranscriptional control of Notch-1.

### siRNA-mediated knockdown of DCAMKL-1 inhibits Epithelial-to-Mesenchymal Transition via a *miR-200a *dependent mechanism

**Figure 5:**
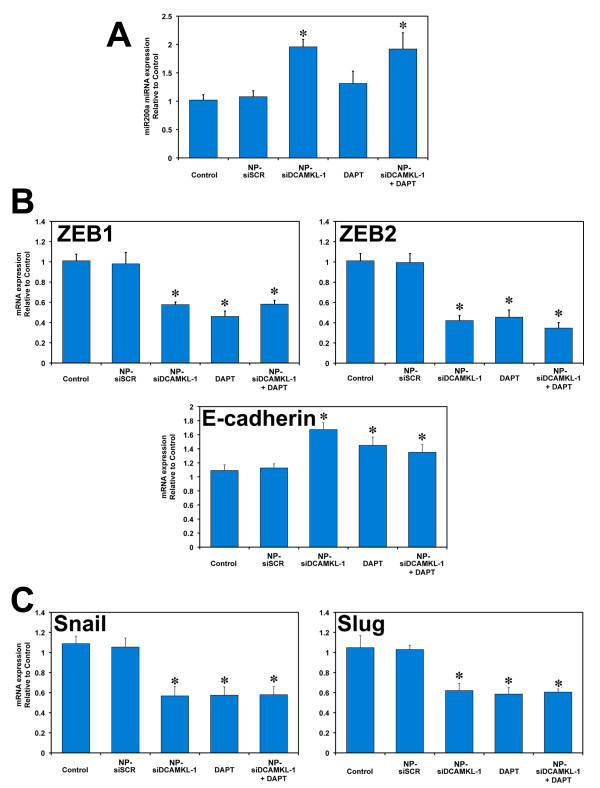
**Inhibition of DCAMKL-1 and Notch signaling inhibits EMT**. (*A*) siRNA-mediated knockdown of DCAMKL-1 in tumor xenografts results in increased expression of *pri-miR-200a *miRNA. Tumor xenografts treated with NP-siDCAMKL-1, DAPT and siDCAMKL-1+DAPT demonstrated a downregulation of EMT transcription factors ZEB1, ZEB2 and increased the expression of E-cadherin mRNA (*B*), decreased snail and slug mRNA expression (*C*). Values in the bar graph are given as average ± SEM, and *asterisks *denote statistically significant differences (*P *< 0.01) compared with control (NP alone).

Epithelial-to-Mesenchymal Transition (EMT) is a phenotypic conversion in fibrotic diseases and neoplasia [33,34]. Recent studies have suggested that mesenchymal gene profiles in tumors are predictive of poor outcome in colorectal, breast, and ovarian cancers [35,36]. Furthermore, recent reports suggest that the downregulation of several miRNAs (*miR-200a*, *miR-200b*, *miR-200c*, *miR-141*, and *miR-429*) is an essential feature of EMT [37]. Consequently, induction of these miRNAs results in inhibition of EMT [37-39]. We subjected the HCT116 tumor xenografts to *miR-200a *miRNA expression analysis by real-time RT-PCR. NP-siRNA-mediated knockdown of DCAMKL-1 resulted in upregulation of *pri-miR-200a *(Figure 5A) and downregulation of ZEB1 and ZEB2 with upregulation of E-cadherin (Figure 5B) in the HCT116 tumor xenografts. We did not observe any difference in expression of *pri-miR-200a *miRNA following treatment with DAPT compared to control or NP-siSCR treated tumors (Figure 5A). These data suggest that Notch-1 inhibition alone was insufficient to induce endogenous *miR-200a *at the dose tested.

We observed a significant downregulation of Snail and Slug in tumor xenografts treated with NP-siDCAMKL-1, DAPT, and NP-siDCAMKL-1+DAPT compared to control or NP-siSCR treated tumors (Figure 5C). These data taken together suggest that knockdown of DCAMKL-1 may inhibit EMT via a *miR-200a*-dependent mechanism in human colorectal cancer [8].

## Discussion

The use of PLGA NPs has emerged as a powerful, potential approach for carrying small and large molecules for therapeutic applications because of PLGA's biodegradability, slow-release of encapsulated payloads and enhanced cellular uptake [40]. PLGA has been approved by the FDA for human use [41]. In this report, we have demonstrated that (a) DCAMKL-1 siRNA encapsulated in PLGA nanoparticles (siDCAMKL-1 NPs) exhibit significant knockdown of DCAMKL-1 mRNA in HCT116 cells; (b) siDCAMKL-1 NPs are similarly potent or more than free liposomal encapsulated siDCAMKL-1 in the ability to down-regulate tumorigenesis, pro-proliferative and oncogenic factors such as c-Myc, in tumor xenografts; (c) siDCAMKL-1 NPs are similarly effective in increasing tumor suppressor miRNA *let-7a*; (d) siDCAMKL-1 NPs are effective in increasing *miR-144 *and downregulating Notch-1 and (e) siDCAMKL-1 NPs may serve as a useful vehicle for the delivery of anti-cancer therapy via its effects on EMT through its interaction with *miR-200a*.

In general, NP-mediated delivery has been considered to enhance the bioavailability of an active component such as a drug, while limiting toxicity. Thus, nanoparticle delivery systems are promising tools for treatment of many diseases including cancer [42,43]. We initially constructed and subsequently characterized PLGA nanoparticles containing siDCAMKL-1 (siDCAMKL-1 NPs) because in PBS at 37°C, they displayed a release profile, characteristic of an initial burst followed by a relatively constant release until day 28 after treatment [44]. This pattern, known as a burst release, is characteristic of hydrophilic drugs encapsulated inside polymeric nanoparticles. The burst release may be explained by the fact that the hydrophilic drug has readily escaped or diffused into the aqueous medium under a concentration gradient. The relatively constant release that follows is primarily due to the hydrolysis of the ester bonds between the individual monomers which results in the degradation of the nanoparticles and hence the sustained release and bioavailability of siDCAMKL-1 NPs [44].

While chitosan (polysaccharide and a cationic natural polymer) has been used for nucleic acids complexation [45], here we chose to utilize the poly-cationic polymer poly (ethylenimine) (PEI) primarily due to its physical structure and potential for a high degree of protonation which aids the PEI-siRNA complex in avoiding degradation [46]. Consequently, we rationalized that the introduction of PEI, into the PLGA matrix could improve the retention of anionic siRNA molecules to encapsulate siRNA molecules in nanoparticles rather than to adsorb on the surface. In addition, PEI is a highly branched cationic polymer that has been used successfully to transfect a variety of cells with relatively low cytotoxicity when complexed with DNA, demonstrating transfection efficiencies significantly better than those seen with other transfection techniques [46,47].

In the efficiency studies comparing siDCAMKL-1 and siDCAMKL-1 NPs, we used equal amounts of free and encapsulated siDCAMKL-1. We calculated the amounts of the encapsulated siDCAMKL-1 to be approximately 7.45 μg siDCAMKL-1 per mg of PLGA NPs. For any amount of free siDCAMKL-1, we used an equal amount of encapsulated siDCAMKL-1 regardless of the amount of the PLGA present. We then, conducted pilot studies to select the most appropriate treatment period of the HCT116 cells with siDCAMKL-1. In these studies, we initially treated the cells for various time periods, ranging from 2 h to 72 h, with various concentrations of siDCAMKL-1 NPs and the equivalent free siDCAMKL-1 concentration. We observed that NP based-siDCAMKL-1 required 7-fold less siRNA to obtain an equivalent downregulation of DCAMKL-1 mRNA.

The Notch signaling pathway is frequently activated in many human cancers [48,49]. NP-based-DCAMKL-1 knockdown in HCT116 tumor xenografts resulted in a marked decrease in Notch-1 mRNA (50%), which contains a putative predicted binding site for *miR-144 *in the 3'UTR. *miR-144 *is a regulator of embryonic *alpha-hemoglobin *(α-*E1*), through targeting the 3'-UTR of *Krüppel-like factor D *gene and positively regulates erythroid differentiation in hematopoietic stem cells [50]. Next we evaluated the expression of *miR-144 *in HCT116 tumor xenografts treated with NP-siDCAMKL-1. There was a 3-fold increase in *miR-144 *and a corresponding ~40% reduction in Notch-1 mRNA and protein, which was further confirmed by the luciferase-based reporter assay.

Pharmacological inhibition of Notch using γ-secretase inhibitor has been demonstrated to block tumor development in various cancers including that of the pancreas [48]. In this report, we have confirmed that DAPT-mediated inhibition of Notch resulted in tumor growth arrest. Given the inhibitory effect of NP-siDCAMKL-1 on Notch, we evaluated the effects of DAPT on *miR-144*. Surprisingly, we observed an 8-fold increase in *miR-144 *and a reduction in DCAMKL-1 mRNA following treatment of tumor xenografts with DAPT. While the exact mechanism is unknown, we speculate that DAPT may act on DCAMKL-1 directly, resulting in the induction of *miR-144*. These data taken together suggest that both DAPT and/or NP-siDCAMKL-1 act on similar pathways. Additionally, the Notch-1 downstream effector HES1 mRNA and protein were decreased following treatment of xenografts with NP-siDCAMKL-1. Here for the first time, we report that DCAMKL-1 regulates Notch-1 via a *miR-144 *dependent mechanism in colorectal cancer.

Recently, inhibition of Notch signaling has been shown to attenuate EMT [51]. In our study, following treatment with DAPT and/or NP-siDCAMKL-1, we observed a reduction in EMT transcription factors Snail, Slug, ZEB1 and ZEB2.

miRNAs have emerged as important developmental regulators and control critical processes such as cell fate determination and cell death [52]. There is increasing evidence that several miRNAs are mutated or poorly expressed in human cancers and may act as tumor suppressors or oncogenes [53,54]. Here we report that NP-siDCAMKL-1 upregulates *miR-200a*, *let-7a *and *miR-144 *in the colorectal cancer tumor xenograft model. Furthermore, induction of *pri-miR-200a *resulted in downregulation of ZEB1, ZEB2, Snail and Slug in colorectal tumor xenografts. These data strongly support a direct regulatory role for DCAMKL-1 in cancer via miRNA dependent mechanisms. As recently reported, the induction of EMT in human mammary epithelial cells resulted in a "stem cell-like" phenotype characterized by a CD44^high ^and CD24^low ^cell surface marker expression pattern. Furthermore, these cells formed mammospheres, colonies in soft agar and tumors in nude mice more aggressively than non-EMT induced cells. These studies demonstrate a direct link between the induction of EMT and the gain of stem cell-like properties [55]. These recent findings lend support to our hypothesis that EMT in the stem cell population may play a critical role in tumorigenesis [8].

Finally, we have shown here that targeting a key regulatory molecule (DCAMKL-1) utilizing NP-based delivery of siRNA results in colorectal cancer tumor xenograft growth arrest through the upregulation of several tumor suppressor miRNAs. Induction of microRNAs that coordinately inhibit critical oncogenic genes could lead to the development of novel anti-cancer therapeutics that attack multiple pathways and processes that are essential for cancer growth, invasion and metastasis in colon and perhaps other cancers.

## Methods

### Reagents

All cell culture reagents were purchased from Sigma Aldrich (St. Louis, MO, USA). PLGA was purchased from Lakeshore Biomaterials (Birmingham, AL, USA) as a 50:50 monomer ratio with a molecular weight of 58 kDa and inherent viscosity of 0.43 dl/g.

### Cell culture

Human colon cancer HCT-116 cells were obtained from the American Type Culture Collection and propogated in Dulbecco's modified Eagle medium supplemented with 10% fetal bovine serum and 1% penicillin-streptomycin in a humidified chamber at 37°C and 5% CO_2_.

### Small interfering RNAs

DCAMKL-1 siRNA (si-DCAMKL-1) sequence targeting the coding region of DCAMKL-1 (accession No. NM_004734) (GGGAGUGAGAACAAUCUACtt) and scrambled siRNAs (si-SCR) not matching any of the human genes were obtained (Ambion Inc, Austin, TX) and transfected using siPORT^™ ^NeoFX^™ ^(Ambion).

### Synthesis and characterization of DCAMKL-1 siRNA NPs

Poly(lactide-*co*-glycolide) acid nanoparticles (PLGA NPs) were synthesized using a double emulsion solvent evaporation technique [44,56]. First siRNA (DCAMKL-1 or scrambled) was condensed on the cationic polymer poly(ethyleneimine) (PEI, 5% w/v) to form an siRNA-PEI complex. siRNA-PEI (200 μl) was added to 30 mg PLGA in 1 ml chloroform (CHCl_3_) and vortexed. This primary emulsion was then transferred into 5 ml of 2% (w/v) polyvinyl alcohol (PVA), which serves as a surfactant, and the entire solution was sonicated on ice for 1 min using a probe sonicator (Misonix XL-2000, Newtown, CT). The organic solvent in the final solution was allowed to evaporate overnight with continuous stirring. NPs were recovered by centrifugation at 20,000 ×*g *for 20 min at 4°C. The supernatant was stored for later assay. The pellet consisting of aggregated NPs was washed three times in water to remove any residual PVA and free, i.e., non-encapsulated, siRNA. NPs were then resuspended in water, freeze-dried for 24 h and then stored at -20°C for later use. The amount of encapsulated siRNA was quantified using a spectrophotometer (DU-800, Beckman Coulter, Brea, CA). The size, polydispersity index, and zeta-potential measurements of synthesized siRNA NPs were determined using diffraction light scattering (DLS) utilizing Zeta PALS (Brookhaven Instruments, Holtsville, NY). Surface morphology of the NPs was examined using a JOEL-JSM-880 scanning electron microscope. Loading efficiency was calculated using the following formula:

$$Loading\;Efficiency\left(\%\right)=\frac{Mass\;of\;siRNA_{NPS}}{Mass\;of\;siRNA_{Tot}}*100$$

where siRNA_NPs _is the amount of siRNA encapsulated inside PLGA NPs, and siRNA_Tot _is the total amount of siRNA added.

### Immunohistochemical analysis

Human multi-cancer tissue microarrays (Tissue Array Network, Rockville, MD) and tumor xenograft tissues were subjected to immunohistochemical analyses. Heat-Induced Epitope Retrieval was performed on 4 μm formalin-fixed paraffin-embedded sections utilizing a pressurized Decloaking Chamber (Biocare Medical) in citrate buffer (pH 6.0) at 99°C for 18 min. **Brightfield**: Slides were incubated in 3% hydrogen peroxide at room temperature for 20 min. After incubation with primary antibody [DCAMKL-1 C-terminal (Abcam Inc., Cambridge, MA) or c-Myc (Santa Cruz Biotechnologies Inc., Santa Cruz, CA) or Notch-1 (Santa Cruz Biotechnologies)], the slides were incubated in peroxidase-conjugated EnVision™+ polymer detection kit (DAKO). Slides were developed with diaminobenzidine (Sigma).

### Microscopic Examination

Slides were examined utilizing a Nikon 80i microscope and DXM1200C camera for brightfield analysis. Fluorescent images were taken with PlanFluoro objectives, utilizing CoolSnap ES2 camera (Photometrics). Images were captured utilizing NIS-Elements software (Nikon).

### Xenograft tumor model

Male athymic nude mice (NCr-nu/nu) were purchased from the National Cancer Institute-Frederick Cancer Research and Development Center (Frederick, MD) and housed in pathogen-free conditions. They were cared for in accordance with guidelines set forth by the American Association for Accreditation of Laboratory Animal Care and the U.S. Public Health Service Commissioned Corps' "Policy on Human Care and Use of Laboratory Animals." All studies were approved and supervised by the Institutional Animal Care and Use Committee. HCT116 cells (6 × 10^6^) were injected subcutaneously into the flanks of 4- to 6-wk-old male athymic nude mice (three mice per group). Tumors were measured using a caliper and the volume was calculated as (length × width^2^) × 0.5. The tumors reached 1000 mm^3 ^15 days after injection of cells. NPs were reconstituted in sterile normal saline and injected directly into the tumors. DAPT was reconstituted in corn oil, which was injected intraperitoneally. In combination treatments, NPs were injected intratumorally and DAPT was injected i.p, at the same time points. Each animal bearing the tumor was injected on days 15, 18, 21, 24, and 27 with one of the following preparations - 50 μl (5 μM) of siRNA-NP preparation [either NP alone (control), NP-siScrambled (siSCR), or NP-siDCAMKL-1], or 10 mg/kg of DAPT alone, or a combination of NP-siDCAMKL-1 and DAPT. All mice were killed on day 30 [9].

### Real-time Reverse Transcription-Polymerase Chain Reaction analyses

Total RNA isolated from tumor xenografts and HCT116 cells was subjected to reverse transcription using Superscript™ II RNase H-Reverse Transcriptase and random hexanucleotide primers (Invitrogen, Carlsbad, CA). The complementary DNA (cDNA) was subsequently used to perform real-time polymerase chain reaction (PCR) by SYBR™ chemistry (SYBR Green I, Molecular Probes, Eugene, OR) for specific transcripts using gene-specific primers and JumpStart™ Taq DNA polymerase (Sigma-Aldrich). The crossing threshold value assessed by real-time PCR was noted for the transcripts and normalized with β-actin messenger RNA (mRNA). The quantitative changes in mRNA were expressed as fold-change relative to control with ± SEM value.

The following primers were used:

β-actin: forward: 5'-GGTGATCCACATCTGCTGGAA-3',

reverse: 5'-ATCATTGCTCCTCCTCAGGG-3';

DCAMKL-1: forward: 5'- CAGCAACCAGGAATGTATTGGA -3',

reverse: 5'- ctcaactcggaatcggaagact-3';

ZEB1: forward: 5'-AAGAATTCACAGTGGAGAGAAGCCA-3',

reverse: 5'-CGTTTCTTGCAGTTTGGGCATT-3';

ZEB2: forward: 5'-AGCCGATCATGGCGGATGGC-3',

reverse: 5'-TTCCTCCTGCTGGGATTGGCTTG-3';

E-cadherin: forward: 5'-CCTCCCATCAGCTGCCC-3',

reverse: 5'-GTGATGCTGTAGAAAACCTT-3';

Snail: forward: 5'-AAGGCCTTCTCTAGGCCCT-3',

reverse: 5'-CGCAGGTTGGAGCGGTCAG-3';

Slug: forward: 5'-TGCTTCAAGGACACATTA-3',

reverse: 5'-CAGTGGTATTTCTTTAC-3';

Twist: forward: 5-GTCTGGAGGATGGAGGG-3,

reverse: 5-TCCTTCTCTGGAAACAATGAC-3;

c-Myc: forward: 5'-CACACATCAGCACAACTACGCA-3',

reverse: 5'-TTGACCCTCTTGGCAGCAG-3';

Notch-1: forward: 5'-CGGGTCCACCAGTTTGAATG-3',

reverse: 5'-GTTGTATTGGTTCGGCACCAT-3'.

### miRNA Analysis

Total RNA isolated from tumor xenografts and HCT116 cancer cells was subjected to reverse transcription with Superscript II RNase H-Reverse Transcriptase and random hexanucleotide primers (Invitrogen). The cDNA was subsequently used to perform real-time PCR by SYBR chemistry for *pri-let-7a, pri-miR-144*, and *pri-miR-200a *transcripts using specific primers and JumpStart Taq DNA polymerase. The crossing threshold value assessed by real-time PCR was noted for *pri-let-7a, pri-miR-144*, and *pri-miR-200a *miRNAs and normalized with *U6 *pri-miRNA. The changes in pri-miRNAs were expressed as fold-change relative to control with ± SEM values [9].

The following primers were used:

*pri-U6*: forward: 5'-CTCGCTTCGGCAGCACA-3',

reverse: 5'-AACGCTTCACGAATTTGCGT-3';

*pri-let-7a*: forward: 5'-GAGGTAGTAGGTTGTATAGTTTAGAA-3',

reverse: 5'-AAAGCTAGGAGGCTGTACA-3';

*pri-miR-144*: forward: 5'-GCTGGGATATCATCATATACTG-3',

reverse: 5'-CGGACTAGTACATCATCTATACTG-3';

*pri-miR-200a*: forward: 5'-TTCCACAGCAGCCCCTG-3',

reverse: 5'-GATGTGCCTCGGTGGTGT-3'.

### Western blot analysis

HCT116 cells or tumor xenograft samples treated with siRNA or siRNA-NPs were lysed and the concentration of protein was determined by the BCA protein assay kit (Pierce Biotechnology Inc., Rockford, IL). Forty μg of the protein was size separated in a 7.5-15% SDS polyacrylamide gel and transferred onto a nitrocellulose membrane with a semidry transfer apparatus (Amersham-Pharmacia, Piscataway, NJ). The membrane was blocked in 5% non-fat dry milk for 1 h and probed overnight with rabbit anti-DCAMKL-1 antibody (Abcam Inc) or with rabbit anti-c-Myc, rabbit anti-Notch1 or rabbit anti-HES1 antibody (Cell Signaling Danvers, MA). Actin, used as a loading control was identified using a goat polyclonal IgG (Santa Cruz Biotechnology Inc). Subsequently, the membrane was incubated with anti-rabbit or anti-goat IgG horseradish peroxidase-conjugated antibodies (Amersham-Pharmacia) for 1 h at room temperature. The proteins were detected using ECL™ Western Blotting detection reagents (Amersham-Pharmacia).

### Luciferase reporter gene assay

HCT116 cells were transfected with a plasmid containing the firefly luciferase (*Photinus pyralis*) gene with a complementary *let-7a (or miR-114) *binding site at its' 3'untranslated region (UTR) obtained from Signosis Inc (Sunnyvale, CA). The cells were also co-transfected with the *Renilla *luciferase expressing plasmid pRL-TK (Promega) as an internal control. Following transfection, the cells were treated with NPs alone, NP-siSCR, or NP-siDCAMKL-1 and subjected to luciferase activity measurement. Luciferase activity was determined as per the manufacturer's instructions (Dual-Luciferase Reporter Assay System; Promega) using a Biotek Synergy III multi plate reader (BioTek, Winooski, VT) as described previously [9]. The activity, normalized to *Renilla *luciferase activity, is presented as relative luciferase units relative to control with ± SEM values. Assays were performed in triplicate wells and experiments were repeated three times.

### Statistical analysis

All experiments were performed in triplicate. Results are reported as average ± SEM unless otherwise indicated. Data were analyzed using the Student's *t*-test. Results were considered statistically significant when *p *< 0.01.

## Competing interests

C.W. Houchen and R.P. Ramanujam are cofounders of COARE Biotechnology, Inc. The other authors disclosed no potential competing interest.

## Authors' contributions

SMS: study concept and design; acquisition of data; analysis and interpretation of data; drafting of the manuscript; critical revision of the manuscript for important intellectual content; statistical analysis and study supervision. RM, FGM, DQ and SP: acquisition of data; analysis and interpretation of data; critical revision of the manuscript for important intellectual content; technical support. PP and SA: analysis and interpretation of data and critical revision of the manuscript for important intellectual content. RPR and CWH: study concept and design; analysis and interpretation of data; critical revision of the manuscript for important intellectual content; obtained funding; material support and study supervision. All authors have read and approved the final manuscript.

## Supplementary Material

Additional file 1 **Figure S1. Downregulation of DCAMKL-1 and Notch signaling decreases tumor xenograft weight**. NP-siDCAMKL-1 and DAPT treatment resulted in significantly decreased tumor weight when compared to control and NP-siSCR treated tumors. Values are given as average ± SEM, and *asterisks *denote statistically significant differences (*P *< 0.01) compared with control (NP alone). Click here for file

Additional file 2 **Figure S2: Notch-1 mRNA has putative binding site for *miR-144***. Representation of the putative binding site for *miR-144 *at 189^th ^base pair position on Notch-1 mRNA 3'UTR (source: http://WWW.microrna.org). Click here for file
