# Supplementary figures and images for: Nanoparticle-based delivery of siDCAMKL-1 increases microRNA-144 and inhibits colorectal cancer tumor growth via a Notch-1 dependent mechanism
Source: J Nanobiotechnology. 2011 Sep 19;9:40. doi: 10.1186/1477-3155-9-40 (PMC3200989; doi:10.1186/1477-3155-9-40)

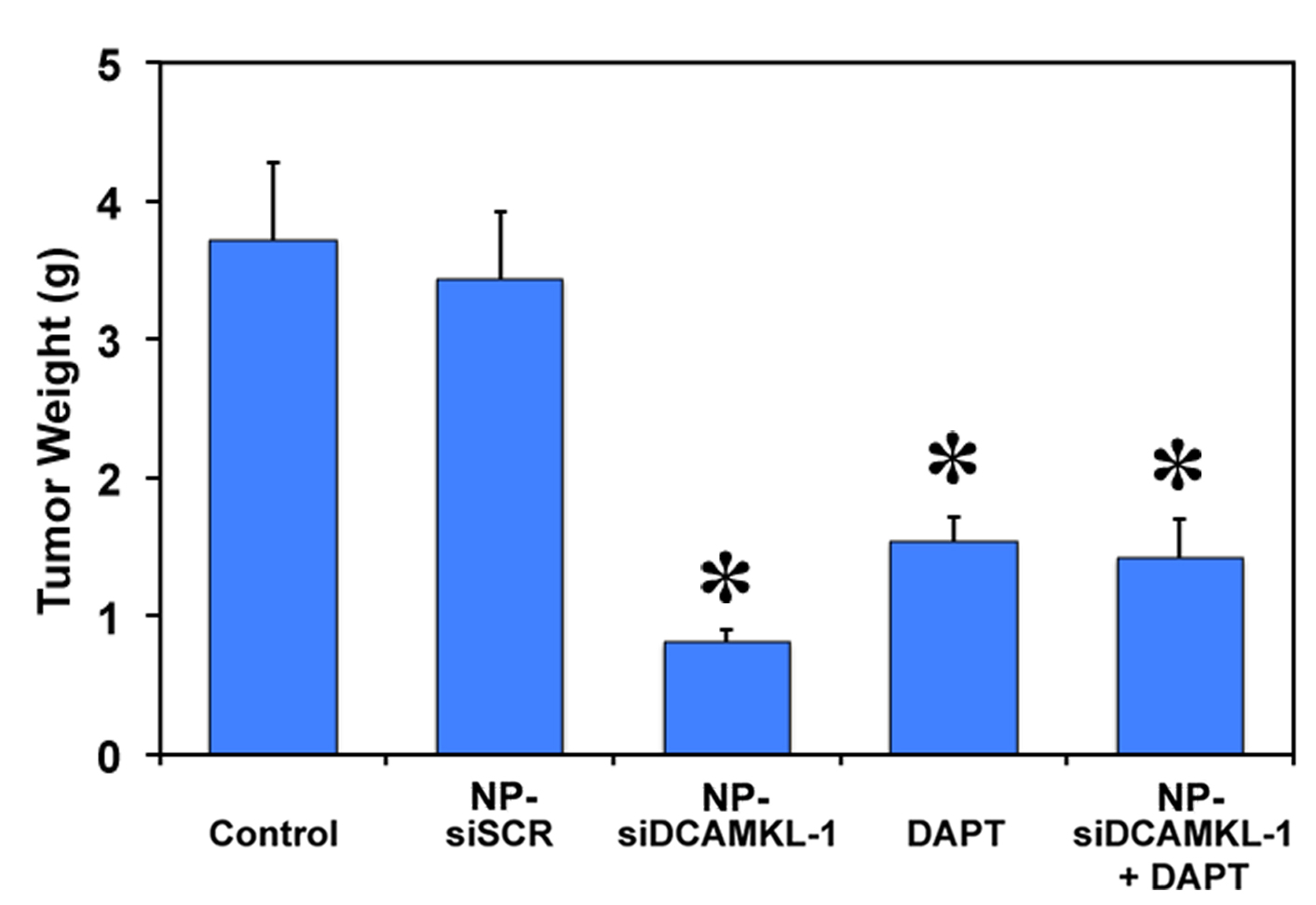

Supplement: Additional file 1 — Figure S1. Downregulation of DCAMKL-1 and Notch signaling decreases tumor xenograft weight. NP-siDCAMKL-1 and DAPT treatment resulted in significantly decreased tumor weight when compared to control and NP-siSCR treated tumors. Values are given as average ± SEM, and asterisks denote statistically significant differences (P < 0.01) compared with control (NP alone). [file 1477-3155-9-40-S1.JPEG]

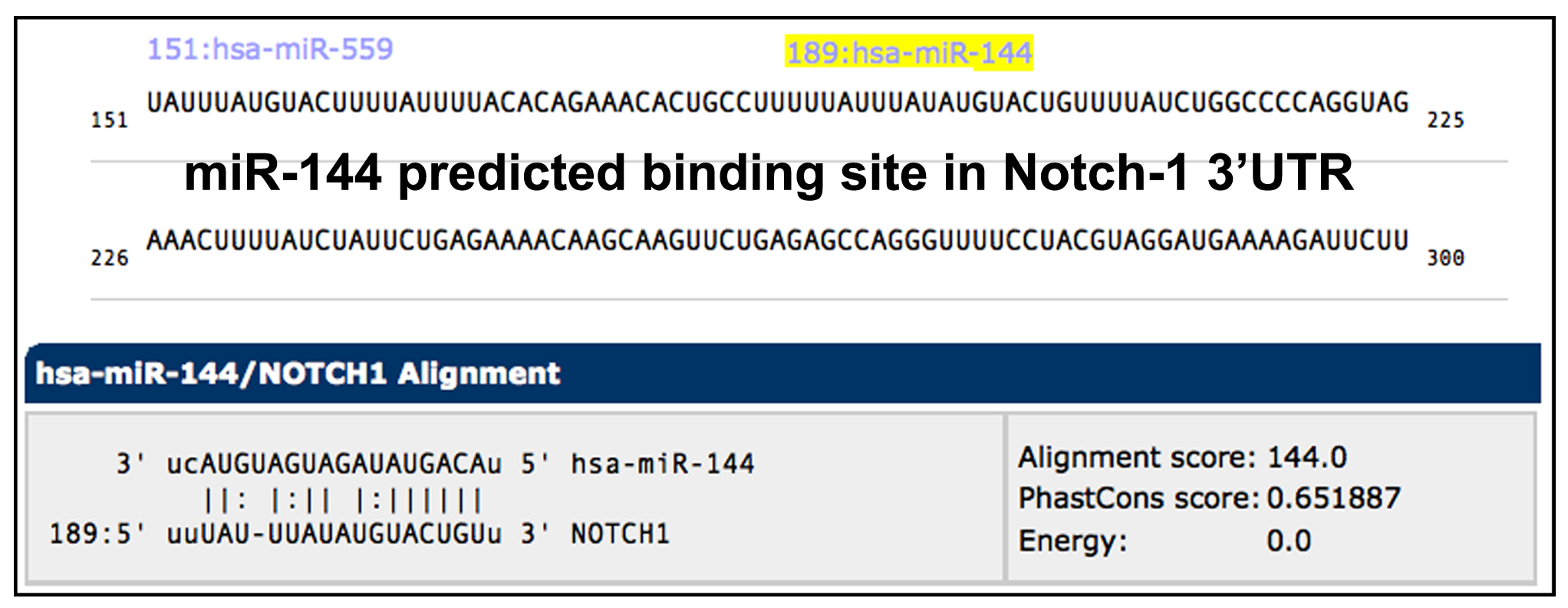

Supplement: Additional file 2 — Figure S2: Notch-1 mRNA has putative binding site for miR-144. Representation of the putative binding site for miR-144 at 189th base pair position on Notch-1 mRNA 3'UTR (source: http://WWW.microrna.org). [file 1477-3155-9-40-S2.JPEG]
